# Epitaxial stabilization and phase instability of VO_2_ polymorphs

**DOI:** 10.1038/srep19621

**Published:** 2016-01-20

**Authors:** Shinbuhm Lee, Ilia N. Ivanov, Jong K. Keum, Ho Nyung Lee

**Affiliations:** 1 Oak Ridge National Laboratory, Oak Ridge, Tennessee 37831, USA

## Abstract

The VO_2_ polymorphs, i.e., VO_2_(A), VO_2_(B), VO_2_(M1) and VO_2_(R), have a wide spectrum of functionalities useful for many potential applications in information and energy technologies. However, synthesis of phase pure materials, especially in thin film forms, has been a challenging task due to the fact that the VO_2_ polymorphs are closely related to each other in a thermodynamic framework. Here, we report epitaxial stabilization of the VO_2_ polymorphs to synthesize high quality single crystalline thin films and study the phase stability of these metastable materials. We selectively deposit all the phases on various perovskite substrates with different crystallographic orientations. By investigating the phase instability, phonon modes and transport behaviours, not only do we find distinctively contrasting physical properties of the VO_2_ polymorphs, but that the polymorphs can be on the verge of phase transitions when heated as low as ~400 °C. Our successful epitaxy of both VO_2_(A) and VO_2_(B) phases, which are rarely studied due to the lack of phase pure materials, will open the door to the fundamental studies of VO_2_ polymorphs for potential applications in advanced electronic and energy devices.

Vanadium dioxides (VO_2_) are strongly correlated *d*^1^ electron systems and are known to have several polymorphs, which include VO_2_(A), VO_2_(B), VO_2_(M1) and VO_2_(R). While the chemical formula is the same, their crystalline and electronic structures are completely different and highly complex, exhibiting many interesting electrical, optical and chemical properties owing to the strong electron correlation^1^,^2^,^3^. Among the aforementioned VO_2_ polymorphs, the rutile VO_2_(R) and the monoclinic VO_2_(M1) have been the most widely studied phases due primarily to their metal-to-insulator transition (MIT) temperature close to room temperature (68 °C)^1^,^2^,^3^. Since this phase transition is accompanied by a huge change in resistivity by three orders of magnitude, VO_2_(R) and VO_2_(M1) have attracted tremendous attention for the electronic and optical applications, such as smart windows^4^, frequency-agile metamaterials^5^,^6^ and electrical switches^7^−^9^.

The monoclinic VO_2_(B) phase has also been explored. However, the focus has been on utilization of the open framework, which originates from the edge-sharing VO_6_ octahedra^10^^−^12. Such open framework makes VO_2_(B) a promising energy material, which can be used as electrodes in Li-ion batteries^13^. Chen *et al.* first reported the growth of textured VO_2_(B) films on (001)-oriented SrTiO_3_ substrates^14^, but pure phase could be stabilized only at thin (<25 nm) films. It is known that the growth of single crystalline VO_2_(B) is very challenging due to the complex crystal structure^14^,^15^. Similarly, the study of the tetragonal VO_2_(A) has so far been very limited^15^−^17^ as compared to other VO_2_ polymorphs, due to the difficulty in synthesizing phase pure crystals. Thus, their physical properties and potential for technical applications have not been much explored.

One of the main reasons for the difficulty in preparing phase pure VO_2_ polymorphs is the narrow range of phase diagram^3^ and, more importantly, the VO_2_ polymorphs are closely related to each other in a thermodynamic framework^10^,^12^,^16^. For example, it has been shown that the VO_2_(A) and VO_2_(B) phases are metastable in bulk and undergo an irreversible phase change into VO_2_(R) upon heating^10^,^12^,^16^, resulting in a mixture of VO_2_ polymorphs. The formation of such mixed phases hinders the accurate understanding of the physical properties of the VO_2_ polymorphs. Hence, preparation of phase pure and high quality crystalline materials has been one of the major challenges in VO_2_ research.

Epitaxial stabilization of crystalline materials by formation of low energy interface is a well-known approach to creating pure phase materials^18^,^19^,^20^,^21^,^22^. Because the stability of these non-equilibrium materials is affected by both thermodynamic and kinetic factors, the highly non-equilibrium film growth conditions offered by pulsed laser epitaxy (PLE) provide a unique opportunity to discover a wide range of materials with unprecedented functionalities.

Here, we report comparatively the physical properties of four VO_2_ polymorphs (i.e., R, M1, A and B phases) epitaxially stabilized by PLE on various perovskite substrates with different crystallographic orientations, i.e., ABO_3_(001), ABO_3_(011) and ABO_3_(111). Distinctively contrasting phase stability, lattice motions and transport properties reported here will provide useful information to develop VO_2_-based electronic devices and energy materials.

## Results and Discussion

In order to selectively grow VO_2_ polymorphs, commercially-available perovskite-oxide substrates, including TbScO_3_ (TSO), SrTiO_3_ (STO), (LaAlO_3_)_0.3_(SrAl_0.5_Ta_0.5_O_3_)_0.7_ (LSAT), LaAlO_3_ (LAO) and YAlO_3_ (YAO), were used. As summarized in Table 1, we were able to epitaxially grow (1) the tetragonal VO_2_(A) phase on (011)-oriented STO and LAO substrates; (2) the monoclinic VO_2_(B) phase on a wide selection of (001)-oriented substrates, including pseudo-cubic TSO, STO, LSAT, LAO and pseudo-cubic YAO; and (3) the monoclinic VO_2_(M1) phase on (111)-oriented STO, LSAT and LAO substrates, which commonly have a 3*m* surface symmetry.

**Figure 1:**
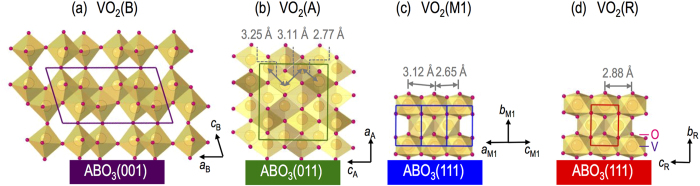
Schematics of (a) VO_2_(B), (b) VO_2_(A), (c) VO_2_(M1) and (d) VO_2_(R) phases grown on various perovskite substrates with different crystallographic orientations, i.e., ABO_3_(001), ABO_3_(011) and ABO_3_(111), respectively.

**Figure 2:**
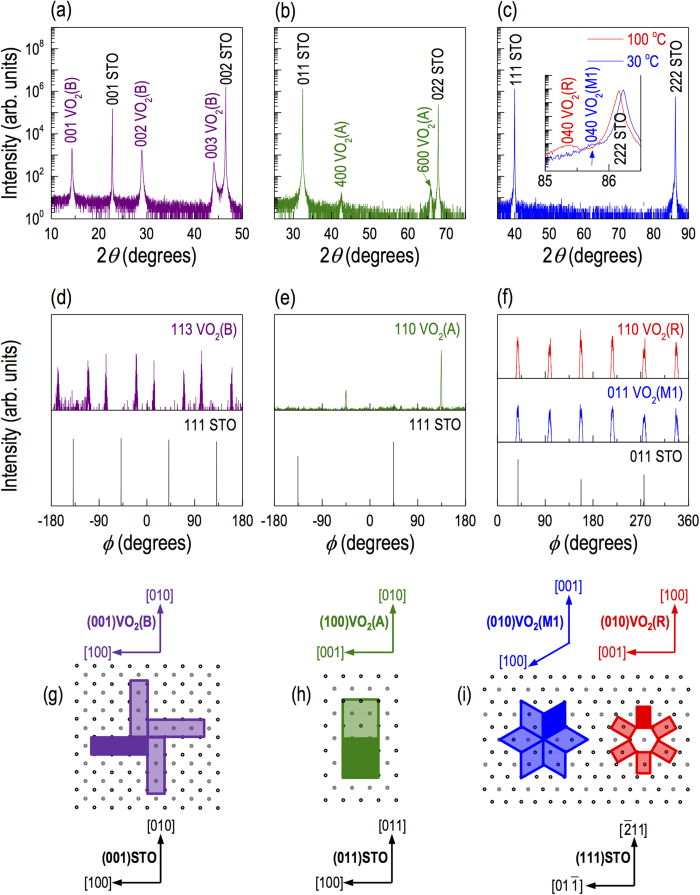
XRD *θ*−2*θ* scans of (a) VO_2_(B), (b) VO_2_(A) and (c) VO_2_(M1) thin films on STO (001), (011) and (111) substrates, respectively. The inset in (c) shows XRD scans of the VO_2_(R) phase (red line) obtained at 100 °C by heating the VO_2_(M1) film (blue line), which is above the *T*_c_ = 68 °C. From *φ* scans shown in (d) VO_2_(B), (e) VO_2_(A) and (f) VO_2_(M1) and VO_2_(R) thin films, in-plane lattice matching is schematically illustrated as shown in (g–i).

The selective growth occurs due to preferential in-plane lattice matching of perovskite-oxide substrates with the VO_2_ polymorphs. VO_2_(B) has a low-symmetry monoclinic structure (space group of C2/m (12)) with lattice constants of *a* = 12.03 Å, *b* = 3.69 Å, *c* = 6.42 Å and *β* = 106.6^o^, as summarized in Table 1 and as schematically shown in Fig. 1a. Various X-ray diffraction (XRD) scans, including *θ*−2*θ* scans shown in Fig. 2a and *ϕ* scans shown in Fig. 2d, for VO_2_(B) films on (001)STO (*a*_STO_ = 3.905 Å) confirmed the following epitaxy relationship: (001)VO_2_(B) || (001)STO and [100]VO_2_(B) || [100]STO (see Fig. 2g). The lattice mismatch 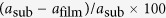 was −2.6% for [010]VO_2_(B) || [010]STO and +5.8% for [100]VO_2_(B) || [100]STO, where the negative and positive signs indicate compressive and tensile strain, respectively.

VO_2_(A) has a tetragonal structure (space group of P4_2_/ncm (138)) with lattice constants of *a* = *b* = 8.43 Å and *c* = 7.68 Å, as schematically shown in Fig. 1b. We found that the single crystalline VO_2_(A) phase could be grown best on (011)STO with the following epitaxy relationship: (100)VO_2_(A) || (011)STO and [010]VO_2_(A) || [011]STO (see Fig. 2h), as confirmed by XRD *θ*−2*θ* scans (see Fig. 2b) and *ϕ* scans (see Fig. 2e). The mismatches along the two orthogonal directions, i.e., [010]VO_2_(A) || [011]STO and [001]VO_2_(A) || [100]STO are −1.7 and +1.7%, respectively.

The VO_2_(M1) phase has a low-symmetry monoclinic structure (space group of P2_1_/c (14)) with lattice constant of *a* = 5.38 Å, *b* = 4.52 Å, *c* = 5.74 Å and *β* = 122.6^o^, as schematically shown in Fig. 1c. There have been several reports on the successful growth of VO_2_(M1) films on substrates with a 3*m* surface symmetry^23^ such as (0001)Al_2_O_3_, (111)MgAl_2_O_4_, (111)MgO and (0001)ZnO. In our study, we mainly attempted to grow epitaxial films on (111)STO substrates to unify the substrates for VO_2_ polymorph films. Since the XRD peak positions of VO_2_(M1) are very close to those of the STO substrate, the film peaks are hardly observed in Fig. 2c. Nevertheless, as confirmed by XRD *ϕ* scans (see Fig. 2f), VO_2_(M1) could be grown on (111)STO with the following epitaxy relationship: (010)VO_2_(M1) || (111)STO and [001]VO_2_(M1) || [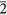
11]STO, as illustrated in Fig. 2i (see the left portion). The lattice mismatch is −3.8% along the [001]VO_2_(M1) || [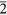
11]STO and +2.6% along the [100]VO_2_(M1) || [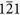]STO.

While the three VO_2_ phases listed above are accessible at room temperature from as grown films, we also tried to access to the VO_2_(R) phase via a structural phase transition by heating a VO_2_(M1) film above the *T*_c_ (68 °C). The VO_2_(R) has a tetragonal structure (space group of P4_2_/mnm (136)) with lattice constants of *a* = *b* = 4.55 Å and *c* = 2.86 Å, as schematically shown in Fig. 1d. As shown in the inset of Fig. 2c, we were able to confirm the phase transition into the VO_2_(R) phase by performing an XRD *θ*−2*θ* scans at 100 °C, which is higher than the *T*_c_. Both VO_2_(R) and VO_2_(M1) phases on (111)STO are (010)-oriented. The epitaxial relationship for VO_2_(R) on (111)STO is illustrated in Fig. 2i (see the right portion) as follows: (010)VO_2_(R) || (111)STO and [100]VO_2_(R) || [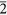
11]STO with the lattice mismatch of +4.9% along the [100]VO_2_(R) || [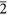
11]STO direction and −3.6% along the [001]VO_2_(R) || [01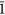]STO direction.

Among the growth parameters, we found that a proper choice of the substrate temperature, *T*_*s*_, is critical, in particular for VO_2_(A) and VO_2_(B) phases on perovskite substrates. As shown in Table 1, we could reproducibly grow VO_2_(A) and VO_2_(B) phases when *T*_*s*_ was lower than 430 °C. On the other hand, the growth of VO_2_(M1) phase was quite insensitive to *T*_*s*_ as we confirmed the growth of high quality films in a wide temperature window (400 ≤ *T*_*s*_ ≤ 600 °C).

**Figure 3:**
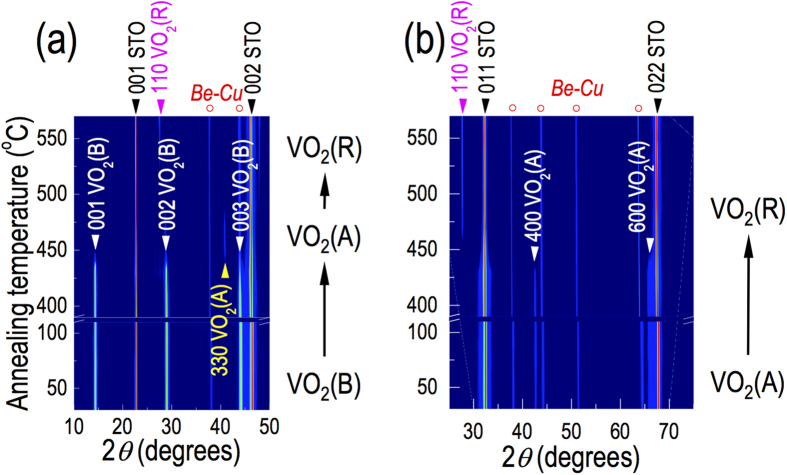
Real time XRD *θ*−2*θ* scans of (a) VO_2_(B)/STO(001) and (b) VO_2_(A)/STO(011) samples as a function of temperature in 0.37 Torr of air. A clear phase change was observed from both samples, indicating that the phases are in close proximity with each other. The phase changes were, however, irreversible upon cooling. It is also worth noting that there are temperature gaps where the XRD peaks are hardly seen before showing up the polycrystalline phases. They are 470–520 °C in (a) and 430–470 °C in (b). We attribute this to the films in metastable state undergoing polycrystallization during the phase transition, even though it is hard to clearly identify due to the suppressed XRD peaks.

To evaluate the thermal stability of VO_2_ polymorphs, epitaxial films of VO_2_(A), VO_2_(B) and VO_2_(M1) phases were heated up to 600 °C. We kept the samples in vacuum (~0.37 Torr) to avoid spontaneous oxidation into the V_2_O_5_ phase^24^. Fig. 3a,b show the phase evolution of VO_2_(B)/STO(001) and VO_2_(A)/STO(011), respectively, characterized by XRD *θ*−2*θ* scans as a function of temperature. In case of VO_2_(B) on STO(001), upon heating, XRD peaks corresponding to (00*l*) VO_2_(B) disappeared above 430 °C and then the (330) VO_2_(A) peak subsequently appeared above 440 °C, indicating the formation of polycrystalline VO_2_(A). When we further increased *T*_*s*_, the VO_2_(A) phase disappeared above 470 °C, and the polycrystalline VO_2_(R) phase appeared above 520 °C. This transformation, i.e., VO_2_(B) → VO_2_(A) → VO_2_(R), indicates that the structural frameworks are similar among the phases. The first transition to A-phase is known to associate with the realignment of VO_6_ octahedra from edge shared to face shared^10^ and, the second transition to the R-phase is attributed to the reorientation of the half of the VO_6_ octahedra^10^.

As shown in Fig. 3b, the VO_2_(A)/STO(011) also revealed similar thermal stability. The peaks corresponding to (*l*00) VO_2_(A) disappeared above 430 °C and polycrystalline VO_2_(R) was subsequently formed above 470 °C. The phase transitions of both VO_2_(B) and VO_2_(A) were irreversible upon cooling. The irreversible phase transformation of VO_2_ polymorphs is similar to what was observed in other binary oxide polymorphs. For example, TiO_2_ is known to undergo a transition from the anatase to the rutile phase via brookite^25^. As the crossover instability in TiO_2_ polymorphs was understood by closely balanced enthalpy among these phases^26^, further thermodynamic studies will be useful to understand the phase instability in VO_2_. The thermal instability of VO_2_(A) and VO_2_(B) explains the formation of mixed phase VO_2_ polymorphs with VO_2_(R) as an impurity phase often observed from films grown above 430 °C. The observation of MIT at 68 °C in VO_2_(A) and VO_2_(B) films grown above 430 °C clearly indicates inclusion of VO_2_(R) as an impurity phase^14^. We note that, on the other hand, the VO_2_(M1) phase was converted into VO_2_(R) at ~68 °C upon heating and was stable up to 600 °C (data not shown). Upon cooling, VO_2_(R) was converted back to VO_2_(M1), indicating a reversible phase evolution with good thermal stability.

**Figure 4:**
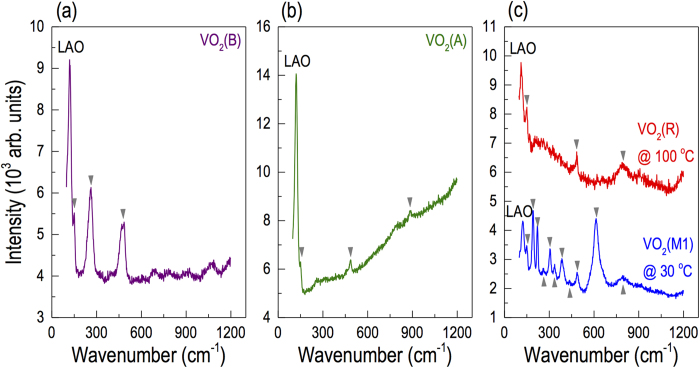
Raman spectra of (a) VO_2_(B), (b) VO_2_(A), (c) VO_2_(M1) and VO_2_(R) grown on LAO substrates. The spectra were recorded at room temperature except the VO_2_(R) phase shown in (c), which was obtained by heating the M1 phase sample to 100 °C in air.

Since the VO_2_ polymorphs have distinct structures, one can expect highly contrasting vibrational characteristics of lattice. Thus, identifying the phonon mode is a good measure of phase purity. In order to comparatively understand the phonon modes, Raman spectroscopy was carried out for the VO_2_ polymorphs by growing films on LAO substrates. The latter were used because dominant Raman spectral features of LAO are isolated at very low wavelength (32 and 123 cm^−1^)^27^. As shown in Fig. 4, the VO_2_ polymorphs revealed contrasting Raman spectra compared to each another. As compared to Raman data available from nanostructured materials^28^,^29^,^30^,^31^, we were able to confirm the phase purity of our epitaxial films.

In addition to the phase confirmation, the Raman spectra from VO_2_ provide more detailed information about the local structure. There are three sets of V−O modes^29^ within wavenumber of 100−1100 cm^−1^. At low wavenumber (<400 cm^−1^), the bands are assigned to V−O−V bending modes; at intermediate wavenumber (400−800 cm^−1^), the bands are attributed to V−O−V stretching modes; and at high wavenumber (>800 cm^−1^), the bands are assigned to V=O stretching modes of distorted octahedra and distorted square-pyramids. As shown in Fig. 4a, the phonon modes in epitaxial films of VO_2_(B) were mainly observed at low and intermediate wavenumbers (152, 263 and 480 cm^−1^), indicating that bending and stretching modes of V−O−V are dominant in VO_2_(B). On the other hand, as shown in Fig. 4b, the phonon modes in VO_2_(A) were mainly observed at high and intermediate wavenumbers (152, 485 and 887 cm^−1^), which imply that the stretching modes of V−O−V and V=O are dominant lattice motions in VO_2_(A). The phonon modes in VO_2_(M1) are very complex and composed of stretching and bending of V−O−V and zigzag chains of V−V. The phonon modes in VO_2_(R) dominantly include stretching modes of V−O−V, which indicate that the crystal structure of VO_2_(R) is more symmetric than VO_2_(M1)^30^,^32^,^33^.

**Figure 5:**
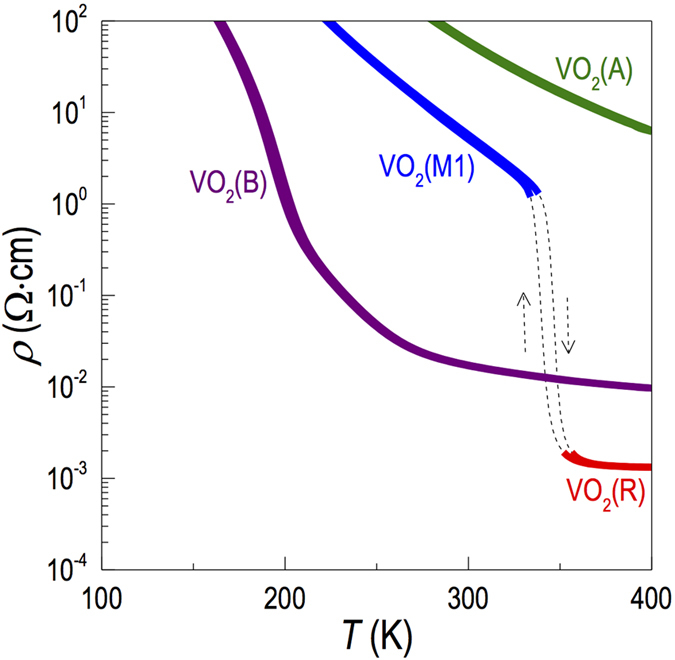
Temperature dependent resistivity for VO_2_(B), VO_2_(A), VO_2_(M1) and VO_2_(R) phases grown on (001), (011), and (111) STO substrates, respectively, exhibiting distinctly contrasting transport behaviours.

While the transport properties of VO_2_(M1) and VO_2_(R) have been extensively studied^1^,^2^,^3^,^8^,^9^,^24^,^32^,^33^,^34^, the physical properties of VO_2_(B) and VO_2_(A) phases have not been much explored due to difficulty in preparing phase pure thin films. Figure 5 shows the transport characteristics of VO_2_(B), VO_2_(A) and VO_2_(M1) films grown on STO substrates. VO_2_(A) showed a monotonic decrease of resistivity as increasing the temperature, typical for insulators. While still insulating over the temperature range we measured, VO_2_(B) revealed more or less semiconducting behaviours with much smaller resistivity compared to that of VO_2_(A), i.e., 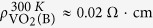 and 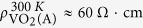. The resistivity in our VO_2_(A)/STO(011) is higher than that reported in VO_2_(A)/STO(001)^15^ by one order of magnitude. The reason is unclear, but one can consider that the film on (001)STO is under a different strain state or that the growth on a (001)STO substrate may include a small amount of VO_2_(B) since their thermal phase boundary is relatively low^10^,^12^,^16^, as shown in Fig. 3. In the case of VO_2_(M1) phase, we also observed the MIT at 340 K from VO_2_(M1) to VO_2_(R) phase change upon heating, similarly observed from many previous studies^1^,^2^,^3^,^8^,^9^,^24^,^32^,^33^,^34^. The MIT accompanied a sudden decrease of the resistivity by 3−4 orders of magnitude, which is comparable to high quality epitaxial films grown on Al_2_O_3_(0001)^24^. This excellent performance could be attributed to the high crystallinity of our epitaxial films (Δ*ω* < 0.1^o^). The transition temperature is consistent with structural phase transition from VO_2_(M1) to VO_2_(R), as shown in XRD *θ*−2*θ* scan in the inset of Fig. 2c. We note that the transport properties of the films grown on LAO substrates were almost identical except for slightly decreased resistivity for films on LAO (data not shown).

Overall, as explained above, the VO_2_ polymorphs revealed a wide range of electronic ground states, i.e., metal [VO_2_(R)], semiconductor [VO_2_(B)] and insulator [VO_2_(A) and VO_2_(M1)], depending on their crystal structure. This wide range of electronic ground states makes VO_2_ highly attractive over other transition metal dioxides, since most other binary oxides are either metal (CrO_2_: α-phase and β-phase) or insulator (TiO_2_: rutile, brookite and anatase). While it is not the main focus of this paper, it is worth mentioning that Goodenough^32^,^34^ obtained a semiempirical expression for the room temperature critical V−V separation *R*_*c*_ ≈ 2.92−2.94 Å for localized and itinerant 3*d* electrons in vanadium oxides.


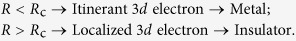


This semiempirical criterion indicates that VO_2_ polymorphs can be either metal or insulator depending on the V−V separation in the distinguishable crystal structures. The VO_2_(R) phase has a uniform V−V separation of *R* = 2.88 Å (ref. 32), resulting in a metallic ground state. As shown in Fig. 1c, the VO_2_(M1) phase has zigzag V−V chains of *R* = 2.65 Å and 3.12 Å (ref. 32). The VO_2_(A) phase also has zigzag V−V chains of *R* = 3.25 Å, 3.11 Å and 2.77 Å (ref. 17) as shown in Fig. 1b. The insulating behaviours that we have observed for those M1 and A-phases are attributed to the localized electrons in shorter V−V chains with *R* = 2.65 Å [VO_2_(M1)] and 2.77 Å [VO_2_(A)]. Thus, overall transport behaviours of our epitaxial thin films can be well explained by Goodenough’s criterion^32^,^34^. Since VO_2_ polymorphs have a wide range of physical properties and, in particular, VO_2_(B) phase is on the verge of becoming a metal, our report on epitaxial synthesis of high quality thin films can open the door to the discovery of novel phenomena and physical properties by deliberate control of the order parameters by various means, including strain, dimensionality, confinement, etc., which can be accessible via epitaxial heterostructuring.

In conclusion, we grew epitaxial films of VO_2_ polymorphs. For the growth of phase pure VO_2_ polymorphs, a careful selection of the growth conditions was necessary especially for the temperature and oxygen pressure. Depending on the crystal orientation of substrates, we found that different phases of VO_2_ could be selectively grown, i.e., VO_2_(B)/ABO_3_(001), VO_2_(A)/ABO_3_(011), VO_2_(M1)/ABO_3_(111), and VO_2_(R)/ABO_3_(111). Such phases revealed unique phonon modes due to the distinctly different crystal structures and physical properties in spite of the same chemical composition. Since the VO_2_ polymorphs have a wide range of electronic ground states from metal [VO_2_(R)] and semiconductor [VO_2_(B)] to insulator [VO_2_(A) and VO_2_(M1)], our epitaxial thin films, which are known to be challenging to grow, will expedite our understanding of underlying physics and developing VO_2_ polymorphs-based electronic devices utilizing the wide selection of the electronic properties from a single composition.

## Methods

### Epitaxial film growth

We deposited epitaxial films (100 nm in thickness) of VO_2_ polymorphs on perovskite oxide substrates by pulsed laser epitaxy. We ablated a sintered VO_2_ target, which contains mainly the M1 phase, by a KrF excimer laser (248 nm in wavelength) at a laser fluence of 1 Jcm^−2^ and at a laser repetition rate of 10 Hz. By growing thin films under a wide range of *P*(O_2_) and *T*_*s*_ (2 mTorr < *P*(O_2_) < 25 mTorr and 350 °C < *T*_*s*_ < 600 °C), we found the optimal condition for VO_2_(A), VO_2_(B) and VO_2_(M1), as described in Table 1. It should be noted that V_2_O_3_ was formed at *P*(O_2_) < 5 mTorr and V_2_O_5_ was formed for *P*(O_2_) > 25 mTorr, due to the multivalent nature of vanadium^24^.

### Characterization of physical properties

To investigate the *dc* transport properties, a physical property measurement system (Quantum Design Inc.) was used with Pt contacts in four-probe geometry. X-ray diffraction (XRD) measurements were carried out with a four-circle high-resolution X-ray diffractometer (X’Pert Pro, Panalytical) using the Cu-K*α*_1_ radiation equipped with a hot stage (DHS 900, Anton Paar). High-temperature environmental XRD measurements were conducted under vacuum with base pressure of 0.37 Torr air. Raman spectra were recorded at various temperatures using a temperature control stage (Lincam Scientific Instruments). A Renishaw 1000 confocal Raman microscope was used to measure Raman spectra in back scattering configuration. Each spectrum is a sum average of seven individual spectra taken at different place on the sample through 20× objective. The wavelength of the Raman laser used in these measurements was 532 nm.

## Additional Information

**How to cite this article**: Lee, S. *et al.* Epitaxial stabilization and phase instability of VO_2_ polymorphs. *Sci. Rep.* **6**, 19621; doi: 10.1038/srep19621 (2016).

## Figures and Tables

**Table 1:** Crystal structure, lattice parameters and growth conditions for VO_2_ polymorphs.

VO_2_polymorphs	Crystal structure (space group)	Lattice constants in bulk				Substrates for epitaxial growth	Critical growth condition
		*a* (Å)	*b* (Å)	*c* (Å)	*β* (^o^)		
VO_2_(A)	Tetragonal (P4_2_/ncm (138))	8.43	8.43	7.68		STO(011), LAO(011)	*T*_*s*_ < 430 °C
VO_2_(B)	Monoclinic (C2/m (12))	12.03	3.69	6.42	106.6	pc-TSO(001), STO(001), LSAT(001), LAO(001), pc-YAO(001)	*T*_*s*_ < 430 °C
VO_2_(M1)	Monoclinic (P2_1_/c (14))	5.38	4.52	5.74	122.6	STO(111), LSAT(111), LAO(111)	Not critical to *T*_*s*_
VO_2_(R)	Tetragonal (P4_2_/mnm (136))	4.55	4.55	2.86		Thermal heating of VO_2_(M1) above 68 °C	
